# Liver transplantation for hepatocellular carcinoma in a patient with hereditary hemorrhagic telangiectasia: a case report

**DOI:** 10.3389/fsurg.2025.1713929

**Published:** 2026-01-09

**Authors:** Jiawei Xu, Decai Yu

**Affiliations:** Division of Hepatobiliary and Transplantation Surgery, Department of General Surgery, Nanjing Drum Tower Hospital, The Affiliated Hospital of Nanjing University Medical School, Nanjing, Jiangsu, China

**Keywords:** liver transplantation, hepatocellular carcinoma (HCC), hereditary hemorrhagic telangiectasia (HHT), ACVRL1, arteriovenous malformation (AVM), case report

## Abstract

**Background:**

Hereditary Hemorrhagic Telangiectasia (HHT) is a rare autosomal dominant vascular disorder causing systemic arteriovenous malformations (AVMs). Hepatocellular carcinoma (HCC) is a common malignancy, and liver transplantation (LT) serves as a curative option for advanced HCC meeting specific criteria. However, the coexistence of HHT and HCC is extremely rare, and HHT-related hepatic vascular abnormalities combined with the risk of AVM recurrence after LT pose unique management challenges. Few cases of LT for HHT-associated HCC have been reported worldwide; herein, we present one successful case.

**Case description:**

A 62-year-old Chinese man was diagnosed with HHT type 2 (harboring an ACVRL1 gene mutation) and Barcelona Clinic Liver Cancer (BCLC) stage B HCC. Preoperative imaging revealed a 55 mm × 32 mm HCC lesion in the right hepatic lobe, along with HHT-related intrahepatic vascular anomalies. He underwent orthotopic LT using a deceased donor liver. Intraoperatively, a “tension-free end-to-end anastomosis” technique was applied to address anomalous hepatic arteries, with real-time Doppler ultrasound monitoring to ensure vascular patency. Postoperatively, an immunosuppressive regimen based on mammalian target of rapamycin (mTOR) inhibitor was administered. The patient recovered uneventfully, with normalized liver function indicators and no evidence of HCC recurrence or HHT-related complications at the 6-month follow-up.

**Conclusions:**

Liver transplantation is safe and effective for HCC complicated by HHT. Successful management relies on comprehensive preoperative evaluation of vascular anomalies and AVMs, intraoperative individualized vascular management, and postoperative immunosuppression combined with close monitoring. Favorable short-term outcomes can be achieved with this strategy.

## 1 Introduction

Hereditary hemorrhagic telangiectasia (HHT) has a global estimated prevalence of 1/5,000–1/8,000. Its core pathogenesis involves mutations in the ENG (encoding the TGF-β type III receptor) or ACVRL1 (ALK1, encoding the TGF-β type I receptor) genes, leading to dysfunction of vascular endothelial cells and subsequent development of systemic capillary telangiectasia and arteriovenous malformations (AVMs) (1). These hepatic AVMs can cause abnormal parenchymal perfusion (hypoperfusion or hyperperfusion) via intrahepatic shunting, potentially inducing nodular hyperplasia [e.g., focal nodular hyperplasia (FNH) or nodular regenerative hyperplasia] (2). However, such nodules are generally benign. While the coexistence of HHT and HCC itself has been sporadically reported (3, 4), the utilization of LT as a curative treatment for such patients is exceedingly rare, with no well-documented cases of successful LT for HHT-associated HCC in the current literature.

As a highly prevalent malignancy worldwide, liver transplantation (LT) serves as a curative treatment for patients with advanced HCC who meet the Milan criteria (5–7). However, the management of patients with both HHT and HCC presents a dual challenge. On one hand, HHT-related hepatic vascular abnormalities (e.g., tortuous and dilated hepatic arteries, anatomical variations in origin) significantly increase the technical difficulty of vascular anastomosis during surgery. On the other hand, HHT patients face a high risk of AVM recurrence post-LT (with a 20-year cumulative recurrence risk of 87%), while also requiring strategies to prevent HCC recurrence, making conventional perioperative protocols insufficient (8). Although the incidence of HCC in HHT patients remains unstudied, chronic regional hypoxia resulting from intrahepatic AVMs may lead to hepatic parenchymal injury and fibrosis, activating hypoxia-inducible factor-1α (HIF-1α)-regulated pathways for angiogenesis and cell proliferation, which could potentially drive hepatocarcinogenesis.

This article reviews the entire LT process for a patient with HHT type 2 and HCC, integrating relevant literature to discuss key aspects of preoperative evaluation, intraoperative techniques, and postoperative management, thereby providing a reference for clinical decision-making in similar rare cases (9).

## 2 Case presentation

### 2.1 Basic information and medical history

A 62-year-old married male, retired. Past medical history included hypertension for 5–6 years, with a highest recorded blood pressure of 150/100 mmHg; he was on long-term oral antihypertensive medication and reported stable blood pressure control. He had a history of hepatic schistosomiasis in childhood, considered clinically cured. No history of hepatitis, tuberculosis, diabetes mellitus, food or drug allergies, or blood transfusions. Surgical history: implantation of a totally implantable infusion pump, hepatic arteriography, and hepatic arterial infusion chemotherapy for hepatocellular carcinoma(BCLC stage B) three months prior. Family history: a first-degree relative (daughter) carried the same ACVRL1 gene mutation as the patient. No history of other genetic diseases or similar conditions in the family.

Physical examination on admission: The patient was conscious, with a slightly poor mental state. No jaundice or significant cutaneous telangiectasia was observed on the skin and mucous membranes (consistent with the clinical feature that some HHT patients lack typical cutaneous manifestations). The abdomen was soft and flat, without abdominal wall venous collaterals; shifting dullness was negative. No edema in the lower limbs. Normal bowel and bladder function, with no significant weight change.

### 2.2 Auxiliary examinations

**Figure 1:**
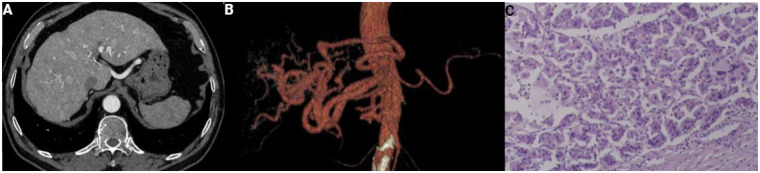
**(A)** axial image of contrast—enhanced liver CT. Tortuous and irregularly distributed vascular malformations in the liver parenchyma induced by Hereditary Hemorrhagic Telangiectasia (HHT) can be observed. **(B)** Three—dimensional reconstructed image of abdominal blood vessels. The tortuous, dilated, and disordered morphological manifestations of hepatic artery branches caused by HHT are clearly demonstrated. **(C)** Postoperative histopathological section (hematoxylin and eosin staining). Vascular malformations around the tumor resulting from HHT can be seen under the microscope.

Imaging and Cardiac Function Assessment: Preoperative non-contrast chest CT showed multiple small nodules in both lungs (largest approx. 3mm × 3 mm, no signs of AVM), mild bronchiectasis with local high-density shadow in the right upper lobe, mildly enlarged mediastinal lymph nodes, cardiomegaly with slight widening of the pulmonary trunk. Hepatic angiography revealed a mass-like slightly hypodense shadow in the right hepatic lobe (max cross-section approx. 55mm × 32 mm), showing heterogeneous enhancement on contrast scan, consistent with HCC characteristics. Also noted were tortuous and dilated left and right hepatic arteries, with a variant origin of the right hepatic artery (arising from the abdominal aorta), multiple slightly hypodense shadows within the liver (considered changes related to schistosomiasis), accompanied by cholelithiasis (stone diameter approx. 0.5 cm). Upper abdominal MRI further confirmed the right hepatic lobe lesion as HCC, with multiple abnormal hyperperfusion areas in the liver (related to HHT vascular malformations). Representative imaging findings are shown in Figure 1. Preoperative cardiac echocardiography showed normal left atrium size (apical four-chamber view 3.8cm × 5.5 cm), normal left ventricular myocardial thickness (basal interventricular septum thickness 1.1 cm), no significant valvular regurgitation, pulmonary artery systolic pressure (PASP) 35 mmHg, left ventricular ejection fraction (LVEF) 63%, with no signs of HHT-related high-output heart failure. Postoperative repeat echocardiography showed mild left atrial enlargement (4.3cm × 6.2 cm), left ventricular myocardial thickening (basal interventricular septum 1.45 cm), mild-to-moderate tricuspid regurgitation, mild mitral and aortic regurgitation, PASP 30 mmHg, LVEF 65.3%, and minimal to small amount of diastolic pericardial effusion, not indicative of HHT-related cardiac function deterioration.

Genetic Testing: Next-generation sequencing identified a heterozygous mutation in the ACVRL1 gene. Sequencing depth 81/71 (0.47). The mutation was located in exon 3, zygosity was heterozygous, and its ACMG classification was Likely Pathogenic (LP). This met the molecular diagnostic criteria for HHT type 2 (autosomal dominant inheritance).

Pathological Examination: The explanted liver specimen revealed hepatocellular carcinoma, histological pattern: compact and trabecular types, without neural invasion. Peri-tumoral liver tissue showed cavernous hemangioma (considered related to HHT vascular abnormalities). No cirrhotic changes were present, only mild fibrosis and vascular dilation (likely related to the combined effects of schistosomiasis history and HHT). Gallbladder tissue showed chronic cholecystitis changes, with a gray-black stone approximately 0.5 cm in diameter in the lumen. Immunohistochemistry results: CK8/18 (3+), CK19 (-), CK7 (-), AFP (focal +), HepPar-1 (partial +), Ki-67 (10%+), HER-2 (focal cytoplasmic +), further supporting the diagnosis of HCC. No specific pathological features suggesting HHT-related vascular malformations involving the tumor tissue were found.

Laboratory Tests (Preoperative): Hemoglobin 10.8 g/dL, platelets 225 × 10³/mm³, International Normalized Ratio (INR) 1.02; Total bilirubin 58 μmol/L, Albumin 35 g/L, ALT 85 U/L, AST 72 U/L, Alkaline Phosphatase 180 U/L; AFP 78 ng/mL, PIVKA-II (DCP) 450 mAU/mL; Hepatitis B surface antigen negative, Hepatitis C antibody negative. No biochemical or imaging evidence of diabetes, fatty liver disease, or iron overload.

### 2.3 Treatment process and follow-up

#### 2.3.1 Preoperative evaluation and indication for LT

The diagnosis of HHT type 2 was confirmed genetically by the identification of a pathogenic ACVRL1gene mutation. The diagnosis of HCC was established based on contrast-enhanced imaging, which revealed a 55 mm × 32 mm mass in the right hepatic lobe, with concomitant elevation of alpha-fetoprotein (AFP, 78 ng/mL). The tumor met the Milan criteria and was classified as BCLC stage B, indicating a clear indication for curative-intent liver transplantation. A comprehensive HHT-specific preoperative assessment was performed. Chest CT and brain MRI effectively ruled out significant pulmonary and cerebral arteriovenous malformations (AVMs). Cardiac echocardiography demonstrated preserved left ventricular function (LVEF 63%) without evidence of HHT-related high-output heart failure. The patient's liver function was Child-Pugh class B. Following this thorough evaluation, which confirmed the absence of absolute contraindications, the patient was deemed a suitable candidate for liver transplantation.

#### 2.3.2 Surgical technique: individualized vascular anastomosis

The patient underwent a classical orthotopic liver transplantation. The surgery was notable for the management of HHT-related hepatic vascular anomalies, specifically a variant origin of the right hepatic artery from the abdominal aorta and significant tortuosity and dilation of both hepatic arteries. To address this technical challenge, a “minimal-tension end-to-end anastomosis” technique was meticulously applied to the hepatic artery to avoid undue traction on the fragile vessel walls. Intraoperative Doppler ultrasound was utilized in real-time to confirm patent blood flow following the anastomosis. Immediate postoperative Doppler ultrasound confirmed excellent blood flow in the portal vein, hepatic artery, and hepatic veins, with no early vascular complications.

#### 2.3.3 Postoperative management and outcomes

A tailored immunosuppressive regimen was initiated, combining a mammalian target of rapamycin (mTOR) inhibitor (everolimus) with tacrolimus and methylprednisolone. The selection of an mTOR inhibitor was strategic, aiming to concurrently prevent allograft rejection and inhibit pathological angiogenesis associated with HHT, thereby potentially reducing the risk of AVM recurrence. Tacrolimus and everolimus trough levels were maintained within target ranges (5–8 ng/mL and 3–5 ng/mL, respectively), and methylprednisolone was successfully tapered off by the fourth postoperative week. The patient's recovery was uneventful, with no episodes of rejection, infection, or vascular thrombosis. Liver function tests normalized promptly: total bilirubin decreased from 58 μmol/L preoperatively to 18 μmol/L by postoperative day 10. The patient was discharged on postoperative day 19. At the 6-month follow-up, graft function remained excellent, and surveillance abdominal ultrasound and tumor markers showed no evidence of HCC recurrence or *de novo* intrahepatic vascular malformations.

## 3 Discussion

This case presents a singular confluence of hepatocellular carcinoma (HCC), type 2 hereditary hemorrhagic telangiectasia (HHT) with an ACVRL1 mutation, and a history of schistosomal liver disease—a combination reported only sporadically worldwide. This rarity is mirrored in the scarcity of epidemiological and mechanistic data; there is a notable lack of cohort studies quantifying HCC risk in HHT populations, and the potential involvement of ENG/ACVRL1 pathways in hepatocarcinogenesis is not yet defined. Clinically, however, this case highlights the necessity of vigilant monitoring. It suggests that in HHT patients, particularly those with concomitant hepatic injuries, AVM-related perfusion abnormalities should be considered a risk factor, necessitating enhanced imaging surveillance for the early detection of nodules, even in non-cirrhotic livers.

Second, HHT-related anatomical variations of hepatic vessels increase surgical difficulty. In this case, there were anomalous origin of the right hepatic artery and tortuosity/dilatation of hepatic arteries. Literature reports indicate that such abnormalities may raise the risk of intraoperative hepatic artery dissection (5%) and postoperative thrombosis (7%) in liver transplantation (LT) (10). Intraoperatively, the adoption of “tension-free anastomosis” technique combined with real-time Doppler ultrasound monitoring successfully avoided vascular complications—this is consistent with previous studies suggesting that HHT patients can safely undergo liver surgery (including LT) provided that large AVMs in the surgical field and severe portal hypertension are excluded preoperatively (9). Additionally, a postoperative immunosuppressive regimen based on mTOR inhibitors not only inhibits abnormal angiogenesis to reduce the risk of AVM recurrence (with a 20-year cumulative recurrence risk of 87%) but also does not increase the recurrence rate of HCC. Thus, this regimen is suitable as a long-term immunosuppressive strategy for HHT patients with concurrent HCC.

The core of preoperative evaluation focuses on comprehensive screening for HHT-related visceral AVMs and cardiac function assessment. Screening for pulmonary and cerebral AVMs is mandatory: there have been previous case reports of fatal postoperative pulmonary hemorrhage due to unrecognized pulmonary AVMs, while cerebral AVMs may increase the risk of postoperative stroke (11). In this case, preoperative chest computed tomography (CT) and cranial magnetic resonance imaging (MRI) ruled out AVMs, and echocardiography was performed to evaluate pulmonary artery pressure and left ventricular function, thereby avoiding the impact of HHT-related high-output heart failure on surgery. If pulmonary AVMs with a diameter >1–3 mm are detected, embolization therapy should be performed prior to LT. For cardiac function assessment, emphasis should be placed on pulmonary artery systolic pressure (normal range: <30 mmHg) and left ventricular ejection fraction (normal range: >50%) (12). In cases of significant cardiac dysfunction, medical management should be initiated first, followed by reassessment of surgical feasibility.

Intraoperative procedures require individualized adjustment of vascular anastomosis strategies. In view of the characteristics of hepatic artery tortuosity, dilatation, or anomalous origin in HHT patients, the principles of “protecting vascular integrity and ensuring unobstructed blood flow” should be followed: excessive traction should be avoided to prevent vascular wall injury; vascular patches may be used for vessels with large diameter discrepancies or thin walls; and real-time Doppler ultrasound should be employed to assess blood flow velocity and direction, with timely adjustment of anastomosis angle. In this case, the anastomosis position and angle were specifically adjusted according to the anomalous origin of the right hepatic artery, and postoperative blood flow monitoring confirmed favorable outcomes.

Postoperatively, a dual prevention and monitoring system targeting both HHT and HCC should be established. For HHT monitoring: abdominal ultrasound should be performed monthly for the first 6 months postoperatively to evaluate vascular flow in the transplanted liver; cardiac function should be assessed every 3 months; and annual chest CT should be conducted to screen for pulmonary AVMs. In the presence of symptoms such as epistaxis or melena, endoscopic examination should be performed promptly, and interventions with tranexamic acid or bevacizumab (if necessary) are recommended. As a vascular endothelial growth factor (VEGF) antibody, bevacizumab not only alleviates HHT-related hepatic AVMs and high-output heart failure but may also serve as a therapeutic option for recurrent HCC (13). For HCC monitoring: tumor markers and contrast-enhanced abdominal MRI should be evaluated every 3 months for the first 2 years postoperatively, and the interval can be extended to every 6 months thereafter to ensure early detection of recurrent signs.

## 4 Conclusion

Liver transplantation is an effective treatment for hepatocellular carcinoma complicated by HHT. The core of management for these patients lies in “comprehensive preoperative screening for HHT-related visceral AVMs and cardiac function assessment, intraoperative tailored management of vascular anatomical variations, and a postoperative regimen incorporating mTOR inhibitors with dual monitoring for AVM and tumor recurrence (14).” Through standardized perioperative management, this case achieved stable graft function and control of HHT. Combined with literature evidence, it further supports the role of the “ischemic cirrhosis theory” in HHT-associated hepatocarcinogenesis and the potential value of anti-angiogenic drugs in recurrence treatment. More clinical studies are needed in the future to provide stronger evidence for the diagnosis and treatment of HHT patients with HCC.

## Data Availability

The original contributions presented in the study are included in the article/Supplementary Material, further inquiries can be directed to the corresponding author.
